# Clinical presentation and imaging results of patients with symptomatic gluteus medius tears

**DOI:** 10.1093/jhps/hnv035

**Published:** 2015-05-19

**Authors:** Dror Lindner, Noam Shohat, Itamar Botser, Gabriel Agar, Benjamin G. Domb

**Affiliations:** 1. Department of Orthopedic Surgery, Assaf Harofeh Medical Center, Zerifin, Israel; 2. American Hip Institute, Chicago, IL, USA; 3. Department of Orthopedics, Stanford University, Stanford, CA, USA; 4. Loyola University Stritch School of Medicine, Chicago, IL, USA; 5. Hinsdale Orthopedics, Hinsdale, IL, USA

## Abstract

Greater trochanteric pain syndrome (GTPS) is a common complaint. Recently, it has become well recognized that tendinopathy and tears of the gluteus medius (GM) are a cause of recalcitrant GTPS. Nevertheless, the clinical syndrome associated with GM tears is not fully characterized. We characterize the clinical history, findings on physical examination, imaging and intraoperative findings associated with symptomatic GM tears. Forty-five patients (47 hips) who underwent GM repair for the diagnosis of tear were evaluated. Pain was estimated on the visual analog scale (VAS) and hip-specific scores were administered to assess functional status. The imaging modalities were reviewed and intra operative findings were recorded. The average patient age was 54 years (17–76), 93% were females. Symptom onset was commonly insidious (75%) and the average time to diagnosis was 28 months (2–240). The most common pain location was the lateral hip (75%). The average pre-surgery VAS and modified Harris Hip Score were 6.65 (0–10) and 55.5 (12–90), respectively. All patients had pathological findings on magnetic resonance angiogram (MRA) ranging from tendinosis to complete tears of the GM tendon. There was a discrepancy between MRA interpretation by a radiologist and findings during surgery. Hip abductor tears are an under-recognized cause of hip pain and hip symptomatology. In this study, we further characterize the clinical presentation of this entity. The data we present here may facilitate early diagnosis, early orthopedic care and avoid unnecessary prolonged patient sufferings.

## INTRODUCTION

Greater trochanteric pain syndrome (GTPS) is a common complaint with an estimated incidence of 1.8 per 1000 persons [1]. Patients usually present with a dull pain on the lateral aspect of the hip, sometimes with radiation posteriorly and into the thigh. The pain is aggravated by pressure on the area, weight bearing and resisted hip abduction.

Tears of the gluteus medius (GM) and gluteus minimus tendons were described by Bunker *et al.* [2] and Kagan [3] in the late 1990s. Each independently coined these tears ‘rotator cuff tears of the hip,’ drawing the analogy to supraspinatus and infraspinatus of the shoulder. Recently, it has become well recognized that tendinopathy and tears of the GM and gluteus minimus tendons are a cause of recalcitrant GTPS [4–11]. Their etiology remains unclear, but similar to rotator cuff tears in the shoulder, tears of the hip abductor tendons seem to occur through a degenerative and progressive process [12]. The true incidence of GM and minimus tears is unknown in the general and athletic population [13].

Although a significant proportion of patients with GTPS will respond to conservative management, with success rates reported at 60–90% [3, 14], a proportion of patients will continue to experience disabling symptoms despite treatment directed at the bursa. Magnetic resonance angiogram (MRA) may be considered for patients with severe pain and hip abductor weakness that is not responsive to these non-surgical measures in order to diagnose GM tendon pathologies or other underlining causes for GTPS [5, 15–18].

**Fig. 1.**
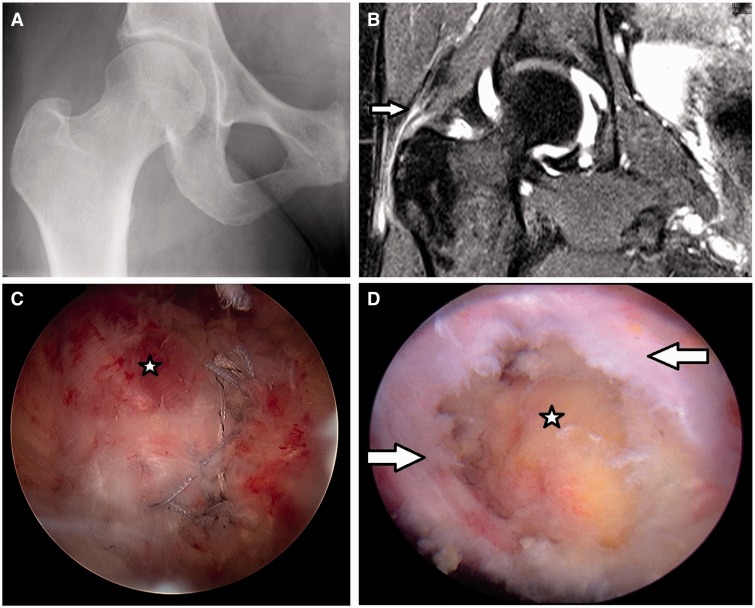
(A) X-ray of a 55–year-old patient with partial GM tear without significant findings; (B) MRA in T2 fat supression protocol of the same patient, arrow pointing to GM tendon partial thickness tears. In that case the radiologist interpeted as resolving tendinosis, while the surgeon as partial GM tear; (D) Arthroscopic image of a debrided partial GM tear, the arrows pointing at the tear, the star located at the bald trochanter; (C) The repaired tendon.

GM tears may be considered as a source for lateral hip pain, yet a definite diagnosis is often delayed [19]. A lack of familiarity with this diagnosis, the absence of X-ray findings and limited clinical information may contribute to this delay (Fig. 1A).

The purpose of this study was to characterize the clinical history, physical examination, imaging findings and intraoperative findings in patients presenting with symptomatic GM tears.

## METHODS

Between February 2008 and November 2011, data were prospectively collected on all patients undergoing hip arthroscopy by the senior surgeon. All patients who displayed a pre-operative painful lateral hip pain or hip abductor weakness, underwent GM repair, were included in the study. Patients with previous hip conditions such as fractures, Legg–Calve–Perthes-Disease, Slipped Capital Femoral Epiphysis and avascular necrosis were excluded.

Data on gender, age, height, weight, body mass index (BMI), duration of symptoms and failure to improve with physiotherapy were also collected. Physical Examination: A detailed physical examination was conducted on all hips prior to surgery. This included passive range of motion (ROM) measurements of flexion, abduction and internal and external rotation. Internal and external rotations were measured while the patient was in a supine position with both the hip and knee flexed at 90°. Tenderness with palpation over the greater trochanter, impingement test of the hip, abductor strength and Trendelenburg sign and gait were examined. The examination was performed and documented by the senior surgeon in a clinical setting. The study was approved by the institutional review board.

The protocol included pre-surgical administration of four hip-specific questionnaires: the modified Harris hip score (mHHS) [20], the non-arthritic hip score (NAHS) [21], the Hip Outcome Score-Activities of Daily Living (HOS-ADL) and the Hip Outcome Score-Sport-Specific Subscale (HOS-SSS) [22]. Patients were also asked to estimate their pain on a visual analog scale (VAS) from 0 to 10, where 0 was considered ‘no pain at all’ and 10 the worst possible pain. These scores were recorded at the pre-operative visit.

### Imaging

MRA was performed on all patients (without the use of anesthesia in the injection) and was evaluated by the senior surgeon and a radiologist; all of the pathologies were documented and reported (Fig. 1B).

### Indications for surgery

Indications for surgery were severe lateral pain interfering with daily living activities or hip abductor weakness and failure to respond to non-operative treatments combined with a pathologic MRI.

### Surgical technique

All arthroscopies were performed by the senior surgeon in the supine position on a traction extension table (Smith & Nephew, Andover,MA). Diagnostic arthroscopy was first performed to check for loose bodies, chondral defects, labral tears, synovitis, ligamentum teres tears and other pathologies. If needed, Cam and Pincer lesions were corrected under fluoroscopic guidance, with acetabuloplasty and femoral osteoplasty, respectively. Labral tears were re-fixated when possible; otherwise, they were selectively debrided until a stable labrum was achieved. Traction was released, and the 70° arthroscope was inserted into the peritrochanteric space through a mid-anterior portal. By aiming just inferior to the vastus ridge under fluoroscopic visualization, the surgeon avoided iatrogenic damage to the GM insertion. A shaver was then introduced through the anterolateral portal. Trochanteric bursectomy was performed, with care to keep the shaver blades away from the GM. When the decision was made to proceed with repair (Fig. 1C), posterolateral and distal peritrochanteric portals were created. These two portals were in line with the center of the trochanter, located 3 cm proximal and 3 cm distal, respectively, to the tip of the trochanter. With the assistance of fluoroscopic guidance, a 5.5-mm Corkscrew anchor (Arthrex, Naples, FL, USA) placed through the tendon split in the distal part of the lateral facet footprint. The Crescent SutureLasso or Birdbeak (Arthrex) was used to pass one limb of each suture through the anterior part of the tendon and one limb of each suture through the posterior part. This was then repeated for a second anchor placed in the more proximal part of the lateral facet. All sutures were tied down by use of an arthroscopic knotting technique (Fig. 1D). Intra-operative data documented included the presence and size of concomitant labral tears, the presence and location of articular cartilage lesions, ligamentum teres tears and GM tears.

### Rehabilitation

All patients undergoing arthroscopic GM suture followed the same rehabilitation protocol. The goals were to protect the repaired tissues, restore ROM, prevent muscular inhibition or gait abnormalities and diminish any pain or inflammation. This was done by first placing patients in a hip brace (Orthomerica, Orlando, FL, USA) for a minimum of 2 weeks after surgery. Patients were restricted to 20 pounds of foot-flat weight bearing activity for 2–4 weeks [23]. The protocol included continuous passive motion for the first 4 weeks. Starting the first day after surgery, patients began stationary biking with a high seat (to avoid pinching) for 2–4 h a day. A slow progression to full strength and activity occurred over a 3–4-month period.

### Statistical analysis

Descriptive statistics was performed using Microsoft Excel (Redmond, WA, USA). Evaluation of agreement between the senior author and radiologist MRA readings was performed using Cohen’s Kappa test.

## RESULTS

### Demographics

Overall 47 hips (45 patients) were included in the study, the average age was 54 years (range 17–76), and 73% (33 patients) were over 50 years old. There was a female dominance, 93% were female. Symptom onset was commonly insidious (75%); however, 12 patients (25%) reported an acute onset of which one had a high energy injury (motor vehicle accident). The average time to diagnosis was 28 months (2–240). The most common pain location was the lateral hip (75%), yet many patients had associated pain in the groin and posterior hip regions (Table I). Three patients (6%) had previous spine surgery, three (6%) patients had prior hip arthroscopy and one patient had prior open hip surgery.

**Table I.** Pain location as described by the patient

Pain location	Number of patients (*N* = 45)	%
Lateral only	19	42
Lateral & Other	37	82
Posterior Hip	16	35
Anterior Hip	13	29
Groin	7	16

### Physical examination

Physical examination demonstrated pain on palpation on the greater trochanter and Trendelenburg sign or gait in the majority of patients. Eighty-three percent of the patients had tenderness with palpation over the greater trochanter, 76% had a positive anterior impingement test, 55% had Trendelenburg gait and 68% had a positive Trendelenburg sign (Table II). Muscle strength was evaluated manually. Thirty patients (64%) had abductor muscle weakness ranging from 4 (18 patients) through 3 (eight patients) and 0–2 (four patients). In the symptomatic hip, average ROM was slightly decreased: flexion was 118 (range 70–135), internal rotation was 26 (range 0–60). The mean VAS was 6.7 (range 0–10) and mHHS was 55.6 (range 12–90) (Table III).

**Table II.** Results of provocative tests

	Positive	Negative	% Positive
Anterior Impingement test	36	11	76
Lateral Impingement test	25	22	53
Posterior Impingement test	15	32	32
FABER test	23	24	49
Trendelenburg test	32	15	68
Trendelenburg gait	26	21	55

**Table III.** Pre-surgery hip scores

	mHHS	HOS-ADL	HOS-SSS	NAHS	VAS
Average	55.6	52.8	26.1	47.7	6.7
Minimum	12.1	14.7	0	0	0
Maximum	90	88.2	75	71.3	10

### Imaging findings

Although all cases included in the study had GM tears confirmed surgically, not all tears were diagnosed on the MRA before the surgery. The radiologist had missed 20 out of 47 cases (43%), diagnosing them as intact GM tendons or tendinosis (Table IV). One patient was interpreted intact by both the senior author and the radiologist; however, a partial thickness GM tear was demonstrated in surgery. When a tear was diagnosed, it was classified as complete or partial. Overall, there was fair agreement between the surgeon and the radiologist with regards to the MRA reading (Kappa 0.31). There was a fair higher agreement between the surgeon and the surgical findings (Kappa 0.35) and a poor agreement between the radiologist and the surgery findings (Kappa 0.14).

**Table IV.** MRA findings according the radiologist and the senior author and the surgical findings

	Radiologist	Surgeon	Surgery
Complete tear	6 (11.8%)	9 (17.6%)	17 (33.3%)
Partial tear	21 (41.2%)	37 (72.5%)	30 (58.8%)
Tendinosis	11 (21.6%)	0 (0%)	0 (0%)
Intact tendon	9 (17.6%)	1 (2%)	0 (0%)
Total	47 (100%)	47 (100%)	47 (100%)

### Surgical findings

A total of 47 GM repairs were evaluated and treated, six were done open and 41 arthroscopically. We used open versus arthroscopic treatment when we believed the tears would be too large for arthroscopic treatment. In the open procedure group five (83%) had full thickness tears and one had high-grade partial thickness tear. In the arthroscopy group, 12 (29%) had full thickness tears and 29 (71%) had partial thickness tears. All the patients in the arthroscopic group had additional procedures; the majority had labral debridement (35 patients) and six had labral repair, whereas only two patients (33%) in the open group had additional procedures (Table V).

**Table V.** Surgical findings

Findings	Open	Arthroscopy	Total
Trochanteric bursitis	3 (50%)	40 (98%)	43 (91%)
GM partial tear	1 (17%)	29 (71%)	30 (64%)
GM complete tear	5 (83%)	12 (29%)	17 (36%)
Labral tear	2 (33%)	41 (100%)	43 (91%)
Cam	0	11 (27%)	11 (23%)
Pincer	0	4 (10%)	4 (8%)
Combined	0	4 (10%)	4 (8%)
Ilio tibial band release	0	2 (5%)	2 (4%)
Ligamentum teres debridement	0	24 (58%)	24 (51%)

## DISCUSSION

GM tears are an under-recognized cause for hip pain and weakness. The clinical presentation of a patient who has a GM tear may vary, and the correct diagnosis may not be considered initially. There is an increase body of literature in regards to imaging modalities for the diagnosis of GM pathologies but only scant reports on treatment and none dedicated to clinical presentation [17, 19, 24–27].

This study provides insight to the diagnosis of GM tears. The average age of our patients undergoing GM repair was 55, similar to other reports. The onset of symptoms of GM tears, while occasionally traumatic or acute, occurred in an insidious fashion in three quarters of our patients. Voos *et al.* [19] in their study of 10 patients undergoing arthroscopic GM had 60% of their patient recall a traumatic event. The largest series was described by Walsh *et al.* [28] reporting on 72 patients undergoing open GM repair, they did not address symptom onset or ROM, all their patients had lateral hip pain and failed conservative treatment.

All patients in this study had either pain on palpation on the greater trochanter or weakness of the abductor muscles. ROM was only slightly decreased when compared with normal ranges. Provocative tests, anterior impingement in particular, were positive in the majority of patients and reduced abductor strength was noticed in 64% of the patients.

Definitive diagnosis of this condition can be difficult as the clinical symptoms and physical findings may vary and subtle. MRI may be used for confirmation of the diagnosis; however, Blankenbaker *et al.* [29] showed that findings of peritrochanteric inflammation on MRI not necessarily correlate with actual disease. In our study, all patients but one had evidence of GM tears on MRA which were confirmed in surgery; however, the MRA was interpreted with no GM tears by a radiologist in 20 patients (42%). We did not assess for false positive; therefore, we can conclude that imaging modalities may aid in the diagnosis but should be evaluated by a physician experienced in treating and diagnosing hip pathologies.

This study had a number of limitations. First, the duration of symptoms was collected retrospectively and could be subject for recall bias. Second, there was no control group of patients with hip pain and no GM pathology; therefore, it is difficult to describe a symptom as pathognomonic for this condition. Third, we cannot exclude the possibility that the symptoms and signs were due to intra-articular pathology such as labral tear or hip impingement.

In our study, we found the average age for GM tears to be 54 years, mostly occurring in females. The average time to diagnosis was 28 months. The most common pain location was the lateral hip, demonstrated on palpation on the greater trochanter and weakness on examination. This pathology can be diagnosed by MRA in a high level of confidence when examined by an experienced physician. The knowledge and awareness of GM tears as a clinical entity is growing. Tears (full thickness or partial) [27] of the GM and minimus tendinous insertions onto the greater trochanter can cause chronic debilitating lateral hip pain. It is therefore paramount that the clinical presentation of GM tears, as outlined in this study, be recognized to establish a timely diagnosis. This will facilitate orthopedic care and avoid unnecessary prolonged patient sufferings.

## FUNDING

Clinic visit, imaging and surgical procedure fees were considered standard of care in this practice and thus were billed to the patients and insurance carriers per standard protocol.

## CONFLICT OF INTEREST STATEMENT

None declared.
